# G Protein-Coupled Receptor and Their Kinases in Cell Biology and Disease

**DOI:** 10.3390/ijms23105501

**Published:** 2022-05-14

**Authors:** Alessandro Cannavo

**Affiliations:** Department of Translational Medical Sciences, Federico II University of Naples, 80131 Naples, Italy; alessandro.cannavo@unina.it

Over the past three decades, after Nobel prizes, Robert Lefkowitz and Brian Kobilka characterized G protein-coupled receptors (GPCRs) structure [1], several clinical and pharmacological evidence advanced our knowledge of how these receptors, and their signaling pathways, influence almost every aspect of the physiology of mammals [2]. Indeed, GPCRs can transduce cellular signals from neurohormones, sensory stimuli, and ions, and their activity is directly modulated by GPCR kinases (GRKs) by phosphorylation that enables the recruitment of β-arrestins (β-Arr1/2) with subsequent desensitization and internalization of the receptor into the endosomes [3,4]. In addition, other regulation mechanisms involve proteins called regulators of G-Protein Signaling (RGS) that bind to and stimulate the GTPase activity with hydrolysis of the active GTP-bound to Gα subunit leading to rapid GPCR signaling turnoff [5]. Nevertheless, a dysregulation of GPCR-signaling along with altered GRKs expression/activity, may induce, or at least influence, the development, and progression of different systemic disorders [6]. Thus, several drugs able to directly inhibit or enhance GPCR signaling have been developed and are currently used in clinical practice [4,6].

The first objective of this Special Issue was to primarily provide an update on the most advanced knowledge and comprehensive elucidation of GPCR function in cell biology. In line with this purpose, the review article from Liu and colleagues [2] discussed the physiological role of GPCRs, along with their related hormones and polypeptides, in disparate organisms, with particular attention to insect physiology. In detail, these authors examine how these receptors are involved in reproduction, development, growth, stress responses, and other biological processes. In addition, they discussed the crucial role of these receptors in insecticide resistance. Continuing with this Topic, Ledonne and coworkers [7] explored the biological function of group 1 metabotropic glutamate receptors (mGluRI), members of the GPCR superfamily, expressed widely in the peripheral and central nervous system and exerting neuromodulatory actions via multiple signaling pathways [8]. In their review article, the authors discussed the interaction between ErbB tyrosine kinases receptors and mGluRI and how these molecules impact processes such as neurotransmission, neuronal excitability, synaptic plasticity, and others [7]. Although this study illustrated the importance of mGluRI, other GPCRs are currently under intensive investigation for their role in regulating some of the processes mentioned above. For instance, among these receptors, hundreds of studies have shown the importance of the growth hormone (GH) secretagogue receptor (GHS-R1a). This receptor is highly expressed in the brain and other peripheral organs/tissues, and together with its ligand, Ghrelin control processes such as neurotransmission and energy metabolism [9]. Of note, a review article from Chihiro Yamada [9] showed how the activity of GHS-R1a receptor and its ligand Ghrelin, along with their involvement in the regulation of physiological processes (e.g., neurotransmission), are involved in regulating energy metabolism. In addition, this author described how gender differences and stress influence GHS-R1a and Ghrelin responsiveness and examined the relationship between GHS-R1a/Ghrelin system and appetite regulation. Finally, Szustak et al. [10], in their original research article, investigated the role of P2Y receptors, a family of purinergic GPCRs activated by nucleotides such as ATP, ADP, UTP, UDP, and UDP-glucose [11], in chondrocytes. In detail, these authors demonstrated that the supplementation of chondrocytes with extracellular nucleotides profoundly impacts their migration and differentiation, advancing the basic understanding of chondrocyte physiology and providing an opportunity to develop a novel strategy to induce cartilage repair [10].

The secondary purpose of this Special Issue was to illustrate and update readers about the latest discoveries linking the role of GPCRs, and their related factors, in different human diseases, hence discussing the therapeutic strategies targeting these factors. In line with this aim, McGowan, and colleagues [12], in their review article, questioned the potential effects of sphingosine (S) kinases, their product S1-phosphate (S1P), and the S1P receptor (S1PRs) targeting in the repertoire of Coronavirus (CoV) disease 2019 (COVID-19) therapies. The clinical presentation of COVID-19 spans from an asymptomatic stage to adverse outcomes or even death [12,13]. These effects are mostly related to an exaggerated immune activation with the release of an array of cytokines and pro-inflammatory and -fibrotic mediators such as interleukins (IL), tumor necrosis factor-alpha (TNFα), Galectin-3 and monocyte chemoattractant protein-1 (MCP1) [12,13]. In response to the severe acute respiratory syndrome (SARS) CoV-2 infection, that causes multi-organ dysfunction, including the lung, brain, and heart. The SK-S1P/S1PRs system controls a variety of physiological roles across a broad range of organisms and exerts beneficial effects on the cardiovascular and nervous systems [14]. In addition, since these molecules are also directly involved in immune and inflammatory responses, acting as positive regulators, the authors discussed throughout the review article the targeting SK-S1P/S1PRs system as a potential strategy to relieve most of the acute and chronic symptoms associated with COVID-19. 

In the context of inflammation, in their original article, Tanner et al. [15] investigated the role of the cytokine IL-6 in the heart. It is well-recognized that following an injury such as myocardial ischemia, the release of this and other cytokines is triggered by immune cells and fibroblasts, leading to excessive myocardial fibrosis. Importantly, these authors demonstrated a new essential activity for β2AR in regulating fibroblast proliferation through Gαs/ERK1/2-induced IL-6 expression/secretion. These findings provide a novel mechanism behind fibroblasts’ response to myocardial injury and expand knowledge around therapies targeting cardiac fibrosis. Interestingly, previous studies attributed part of the effects mentioned above to the Gαs-dependent activation of p-38 mitogen-activated protein kinase (MAPK). p-38 MAPK family are critical regulators of cell signaling (MAP), controlling many physiological and pathological conditions. Notably, many of these processes are regulated by the MKK3 and 6 protein kinases that are well-known activators of p-38. However, despite decades of studies investigating novel therapeutic strategies impacting this p-38-signaling pathway, significant gaps remain. Therefore, in a review article published in this issue, Burton et al. [16] explored the role of the so-called "atypical" p-38 signaling pathway, which is an MKK3/6 independent and alternative pathway of activation of p-38. Importantly, in this study, the authors depicted the mechanism of activation of this atypical signaling pathway. In addition, they provided several pieces of evidence supporting the importance of developing selective inhibitors to hamper this atypical p38 signaling in a wide array of diseases such as dermal and vascular inflammation, cancer, myocardial ischemia, and diabetes complications during pregnancy, bacterial and viral infections. Given the importance of the biological role of GPCRs, it is not surprising that their direct regulators called GPCR kinases (GRKs) are similarly involved in various physiological and pathophysiological processes. There are three main types of GRKs: rhodopsin/visual kinases (GRK1 and 7), the β-adrenergic receptor kinases (βARK1 [GRK2] and βARK2 [GRK3]), and the GRK4 subfamily (GRK4, 5 and 6). Chaudhary and Kim [3], in their review article, summarized most of the GRKs-related activities within the cells and explored their implication in different disorders, with a particular emphasis on thrombosis and hemostasis. However, among the big family of GRKs, GRK2 and 5 are ubiquitously expressed and are of particular importance because of their multiple functions and their role in the pathogenesis of chronic-degenerative disorders including cancer, cardiovascular, and neurological diseases, that mostly depends on their subcellular location [4,17]. Importantly, Marzano and coworkers [6] in their perspective article, reviewed the novel research linking GRK5 to these diseases and the current state-of-the-art of targeting GRK5 as a therapeutic strategy to treat these disorders, while the study from Kayki-Mutlu and Koch [18] reviewed the importance GRK2 and β-arrestins (β-Arr1/2) in nitric oxide (NO)-dependent effects. The NO is a gasotransmitter involved in many complex reactions and mediates numerous physiological functions in the cardiovascular, nervous, and immune systems. Part of these effects is mediated by a reaction between NO and a thiol (-SH) group of a cysteine residue of different proteins, including GRK2 and β-Arr1/2. Of note, S-nitrosylation of GRK2 elicits an inhibitory effect limiting the ability of this protein to phosphorylate and desensitize some GPCRs like the β2AR with important beneficial effects in the cardiovascular system.

In conclusion, overall, the exciting contributions of this Special Issue expanded our knowledge about GPCRs and GRKs biological functions. In addition, all the evidence provided above opens the path to developing a novel class of pharmacological and non-pharmacological entities targeting GRKs, GPCRs, and their related molecules that may offer benefits against various human diseases, including cardiovascular, neurodegenerative, and neoplastic disorders.

## References

[1] Benovic J.L. (2012). G-protein-coupled receptors signal victory. Cell.

[2] Liu N., Wang Y., Li T., Feng X. (2021). G-Protein Coupled Receptors (GPCRs): Signaling Pathways, Characterization, and Functions in Insect Physiology and Toxicology. Int. J. Mol. Sci..

[3] Chaudhary P.K., Kim S. (2021). The GRKs Reactome: Role in Cell Biology and Pathology. Int. J. Mol. Sci..

[4] Cannavo A., Komici K., Bencivenga L., D’amico M.L., Gambino G., Liccardo D., Ferrara N., Rengo G. (2018). GRK2 as a therapeutic target for heart failure. Expert Opin. Ther. Targets.

[5] Tobin A.B. (2008). G-protein-coupled receptor phosphorylation: Where, when and by whom. Br. J. Pharmacol..

[6] Marzano F., Rapacciuolo A., Ferrara N., Rengo G., Koch W.J., Cannavo A. (2021). Targeting GRK5 for Treating Chronic Degenerative Diseases. Int. J. Mol. Sci..

[7] Ledonne A., Mercuri N.B. (2020). Insights on the Functional Interaction between Group 1 Metabotropic Glutamate Receptors (mGluRI) and ErbB Receptors. Int. J. Mol. Sci..

[8] Ferraguti F., Crepaldi L., Nicoletti F. (2008). Metabotropic glutamate 1 receptor: Current concepts and perspectives. Pharmacol. Rev..

[9] Yamada C. (2021). Relationship between Orexigenic Peptide Ghrelin Signal, Gender Difference and Disease. Int. J. Mol. Sci..

[10] Szustak M., Gendaszewska-Darmach E. (2020). Extracellular Nucleotides Selectively Induce Migration of Chondrocytes and Expression of Type II Collagen. Int. J. Mol. Sci..

[11] Doze V.A., Perez D.M. (2013). GPCRs in stem cell function. Prog. Mol. Biol. Transl. Sci..

[12] McGowan E.M., Haddadi N., Nassif N.T., Lin Y. (2020). Targeting the SphK-S1P-SIPR Pathway as a Potential Therapeutic Approach for COVID-19. Int J. Mol. Sci..

[13] Cannavo A., Liccardo D., Gelzo M., Amato F., Gentile I., Pinchera B., Femminella G.D., Parrella R., DERosa A., Gambino G. (2022). Serum Galectin-3 and Aldosterone: Potential biomarkers of cardiac complications in patients with COVID-19. Minerva Endocrinol..

[14] Arosio B., Corbi G., Davinelli S., Giordano V., Liccardo D., Rapacciuolo A., Cannavo A. (2022). Sex Differences in Cardiovascular Diseases: A Matter of Estrogens, Ceramides, and Sphingosine 1-Phosphate. Int. J. Mol. Sci..

[15] Tanner M.A., Thomas T.P., Maitz C.A., Grisanti L.A. (2020). β2-Adrenergic Receptors Increase Cardiac Fibroblast Proliferation Through the Gαs/ERK1/2-Dependent Secretion of Interleukin-6. Int. J. Mol. Sci..

[16] Burton J.C., Antoniades W., Okalova J., Roos M.M., Grimsey N.J. (2021). Atypical p38 Signaling, Activation, and Implications for Disease. Int. J. Mol. Sci..

[17] Marzano F., Liccardo D., Elia A., Mucio I., de Lucia C., Lucchese A.M., Gao E., Ferrara N., Rapacciuolo A., Paolocci N. (2022). Genetic Catalytic Inactivation of GRK5 Impairs Cardiac Function in Mice Via Dysregulated P53 Levels. JACC Basic Transl. Sci..

[18] Kayki-Mutlu G., Koch W.J. (2021). Nitric Oxide and S-Nitrosylation in Cardiac Regulation: G Protein-Coupled Receptor Kinase-2 and β-Arrestins as Targets. Int. J. Mol. Sci..

